# Spatial Multicriteria Decision Analysis of Flood Risks in Aging-Dam Management in China: A Framework and Case Study

**DOI:** 10.3390/ijerph8051368

**Published:** 2011-05-04

**Authors:** Meng Yang, Xin Qian, Yuchao Zhang, Jinbao Sheng, Dengle Shen, Yi Ge

**Affiliations:** 1 State Key Laboratory of Pollution Control and Resource Reuse, School of the Environment, Nanjing University, Nanjing 210046, China; E-Mails: yngjyangmeng@gmail.com (M.Y.); ge.ellie@gmail.com (Y.G.); 2 Dam Safety Management Department, the Ministry of Water Resources of China, Nanjing 210029, China; E-Mail: jbsheng@nhri.cn; 3 Hydraulic Investigations and Design Institute of Chuzhou, Chuzhou 239000, China; E-Mail: sdl0308@sohu.com

**Keywords:** aging dams, flood-risk analysis, dam removal, dam decommissioning

## Abstract

Approximately 30,000 dams in China are aging and are considered to be high-level risks. Developing a framework for analyzing spatial multicriteria flood risk is crucial to ranking management scenarios for these dams, especially in densely populated areas. Based on the theories of spatial multicriteria decision analysis, this report generalizes a framework consisting of scenario definition, problem structuring, criteria construction, spatial quantification of criteria, criteria weighting, decision rules, sensitivity analyses, and scenario appraisal. The framework is presented in detail by using a case study to rank dam rehabilitation, decommissioning and existing-condition scenarios. The results show that there was a serious inundation, and that a dam rehabilitation scenario could reduce the multicriteria flood risk by 0.25 in the most affected areas; this indicates a mean risk decrease of less than 23%. Although increased risk (<0.20) was found for some residential and commercial buildings, if the dam were to be decommissioned, the mean risk would not be greater than the current existing risk, indicating that the dam rehabilitation scenario had a higher rank for decreasing the flood risk than the decommissioning scenario, but that dam rehabilitation alone might be of little help in abating flood risk. With adjustments and improvement to the specific methods (according to the circumstances and available data) this framework may be applied to other sites.

## 1. Introduction

There are more than 85,000 dams in China. Most were built during the period between the 1950s and the 1970s, and an increasing proportion are approaching or exceeding their designed lifespans [1]. Approximately 30,000 of them (36%) are considered to be of high-level risk because of structural obsolescence and/or lack of proper maintenance [2]. Those aging dams that do not satisfy current flood or other loading criteria and do not adhere to current state-of-the-art practices pose significant risks (e.g., risks of property damage and loss of life if the dam fails) to downstream regions, especially in highly populated areas.

Dam rehabilitation is the predominant scenario for resolving this problem in China [3]. However, it is impossible to reinforce all dams in China within a short time under the current economic conditions. In addition, as societal values change, some dams become undesirable from an economic, environmental, public interest, or political point of view. When any dam becomes a hazard that is uneconomical to repair or is deemed undesirable, treatment alternatives may include decommissioning. Internationally, dam decommissioning is being increasingly considered and this reflects a growing concern over dams’ adverse ecological, social, and economic impacts [4,5]. While there were more than 1,600 dams that were decommissioned before the end of 2002 in China [1], increased attention has been paid to decommissioning since *The Management Regulations of Dam Degrading and Decommissioning (Trial)* was promulgated by the Ministry of Water Resources of China (MWR) in 2003.

In fact, decommissioning is not widely accepted by water resource managers in China who worry about the potential increase in flood risk after a structure is decommissioned. In addition, there is no formal framework or guidelines for analyzing the changes of flood risks in that occur during aging-dam management. As a result, the progress of implementation of aging-dam management (especially for small and medium-sized dams) in China is slow. Furthermore, the effects of decommissioning on flood risk have not been adequately considered. Only a few examples that evaluated the impacts of dam removal on hydrology (rather than flood risk) can be found in the literature [6-9]. Developing a framework for flood-risk analysis is crucial for decision making in the management of aging dams. In the domain of dam safety management, risk mainly refers to the probability of dam failure (such as in the studies of Kuo *et al.* [10] and Kwon *et al.* [11]). However, flood-risk analyses encompass the hazard flood event and its possible consequences. This report attempts to address this gap by a using spatial multiple-criteria framework for flood-risk analysis.

Decision making for water-management scenarios regarding flood risk involves multiple criteria, including economic, social and environmental criteria [12,13]. Decision makers must also locate information about changes in spatial flood risk for identifying local hotspots and for developing local immigration measures [14]. Spatial multicriteria decision analysis (MCDA) is useful for managing flood risk. MCDA is defined as a collection of techniques for analyzing geographic events for which the results of the analysis depend on the spatial arrangement of events [15]. There are few studies that have used spatial MCDA in the field of flood-risk management and analysis. These studies were not completely consistent with MCDA theory, or they had objectives different from aging-dam management. For example, Kubal *et al.* [16] assigned a weight of 0.9 out of 1 to environmental criteria without explaining why they chose such an extreme value, whereas Meyer *et al.* [17] gave weight to indicators that only considered the decision-makers’ objectives. Raaijmakers [18] evaluated land-use alternatives based on their suitability as flood-risk management alternatives for coastal zones where the flood-management objectives are different from those for areas downstream of the dam sites. Based on spatial MCDA theory, the research discussed here first introduces a general framework for the spatial analysis of changes in the multicriteria flood risk of aging-dam-management scenarios; the framework is then applied to a case study.

## 2. The General Framework

**Figure 1.**
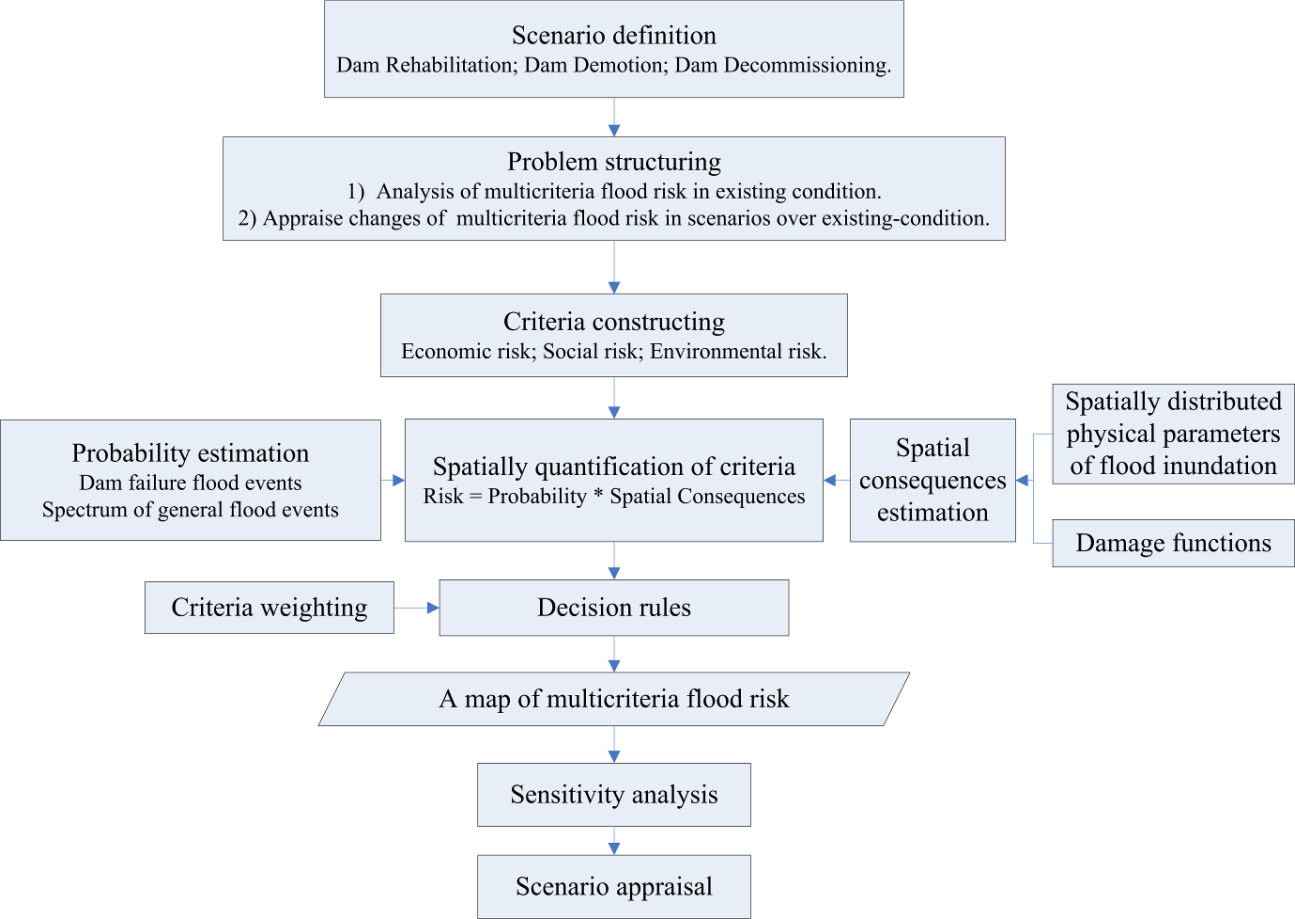
The general procedure of spatial multicriteria flood-risk analysis in the management of aging dams.

Based on spatial MCDA theory [15], the major components of the framework are generalized in Figure 1. The framework mainly consists of the following eight steps: scenario definition, problem structuring, criteria construction, spatial quantification of criteria, criteria weighting, decision rules, sensitivity analyses, and scenario appraisal.

In the context of aging dam management, there are three scenario categories: dam rehabilitation, demotion, and decommissioning. Dam rehabilitation refers to repairing or strengthening of the dam and appurtenant structures to reach an acceptable level of safety and function while not significantly lowering the original design rank [3]. Demotion means reduction in the design rank of the dam so that it still retains some economic benefits (on the premise that an acceptable safety level is achieved). Decommissioning is defined as the full or partial removal of an existing dam and its associated facilities or significant changes to the operations thereof.

The problem of flood-risk analysis is defined as using the existing condition as the benchmark to identify how the multicriteria flood risk of each decision scenario might be changed. It can be structured into two steps. The first step is a multicriteria flood-risk analysis, which is an important prerequisite of step 2 [19]. Associated with operating an existing system, the problem is to identify the magnitude and spatial distribution of flood risk and to determine areas where the risk is high and vulnerable to system changes. When an existing system can no longer adequately meet the needs of the community because of dam aging, new requirements for the dam, changes in land use, or an increase in public safety perceptions, then the next set of actions begins: planning for a revised or rehabilitated system that is adapted to the altered conditions. The second step is a multicriteria scenario appraisal to identify the scenario that is best adapted to the new condition. If this scenario cannot reduce the flood risk, at least it does not increase it.

The appraisal criteria are the changes in flood risks, and they include economic, social and environmental dimensions. The relevant literature is examined to obtain an overview of what criteria should be used to analyze the multicriteria flood risk. The indicators are then constructed following the five desirable properties of criteria, namely criteria that are unambiguous, comprehensive, direct, operational, and understandable [20].

The spatial quantification of each criterion consists of the mapping of its flood risk. Flood risk is defined as a function of the probabilities and spatial consequences of flood events [21]; these events consist of dam failure and a spectrum of general flood events. Spatial consequences are the combined results of the spatial distribution of the physical characteristics of flood inundation (i.e., depth, velocity, and duration) and damage functions. After quantification of the criteria, criteria weights are obtained by considering the opinions of decision makers and relevant stakeholders. Using specific decision rules, the geographical data and criteria weights are combined and transformed into a map of multicriteria flood risk.

A sensitivity analysis is carried out to identify the effects of changes in the input (geographical data and stakeholders’ preferences) on the outputs (ranking of scenarios). The last step of the framework is apprising scenarios for providing recommendations for aging-dam management.

## 3. Case Study

### 3.1. The Study Area and Scenario Definition

**Figure 2.**
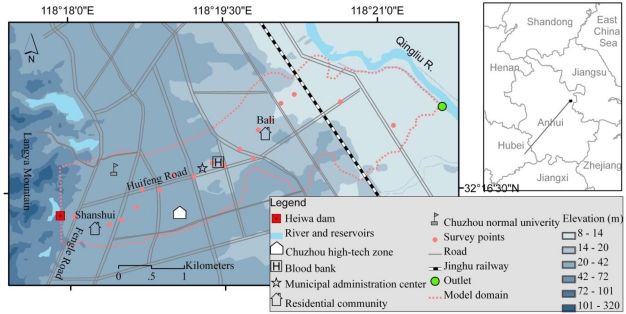
Study area and locations of important elements vulnerable to flood damage.

The study area was the southwest region of Chuzhou City, which is situated at 32.3° N, 118.5° E in Anhui Province, China. The area slopes from Langya Mountain in the southwest to the Qingliu River (Figure 2), and changes topographically from mountainous terrain to flat land. It is located in the East Asian monsoon zone and has an annual average temperature of 15.2 °C and an annual average precipitation of 1,034 mm (57% of which occurs between June and August). There was a small channel that drained water from the upper mountain area to the Qingliu River. During the progress of city expansion, the natural channel was mostly encroached upon, thus compounding flooding problems. The Heiwa Dam, located at the foot of Langya Mountain (Figure 2), is an earthen dam built in 1950 for irrigation and mountain flood control. Owing to the poor construction quality and management, the dam has aged and deteriorated over the past 60 years. It is no longer operated for its original purpose because the surrounding farmlands have been largely converted to urban areas. The dam site and its surrounding areas have been allocated to the construction of a new campus of Chuzhou Normal University.

Two management scenarios were proposed for the case study of the Heiwa Dam (Table 1). The first one is a dam-decommissioning scenario that would partially remove the dam and retain only a small portion of the reservoir storage capacity for recreational purposes.

Through decreasing the dam height to 4 m, this scenario is expected to significantly decrease the probability of dam failure. There will however be no storage capacity for future flood control if this scenario is adopted, so there is concern that the flood risk may increase under this scenario. The second scenario is dam rehabilitation, which refers to repairing the dam and its associated structures to achieve an acceptable level of safety. In addition, through decreasing the normal operating depth by 1.2 m, part of the storage capacity would be adjusted for flood control. As a result, the storage capacity for flood control would be increased by 45%.

### 3.2. Criteria Construction

**Figure 3.**
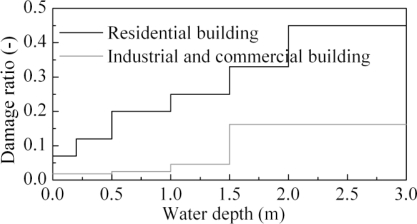
Depth-damage curves of residential, industrial and commercial buildings.

The study area is densely populated. The criteria were constructed according to a similar study [16] and are listed in Table 2. The direct economic loss to assets and inventories of residential, industrial and commercial buildings was used as an indicator of economic loss. The asset values that are at risk were estimated based on official statistics [22]. The value of a unit area was 652 yuan/m^2^ for residential buildings and 640 yuan/m^2^ for industrial and commercial buildings. Depth-damage curves were used to calculate the damaged share of these values. The depth-damage curves in this study (Figure 3) were extrapolated from other sites in China [23,24] and attention was paid to the similarity in building type and contents during the extrapolation [25].

Another economic indicator was aggregated economic risk, which was based on the types of infrastructure (including railways, telephones and electricity; see Table 2). A binary approach [16] was applied that calculated damage using simple Boolean 0 and 1 values to spatially allocate the elements of risk,. If a risk cell was inundated, the damage was set to 1, which indicates that the presence of the element in the flood zone increased the flood risk; otherwise, the damage was set to 0. Although this approach has a higher uncertainty and does not account for the inundation depth, it can simplify the process of locating elements at risk in a complex system with limited available data [16].

The environmental damage caused by flood events should not be ignored, especially in developing countries that are in the process of urbanization and have large areas of construction sites. These sites are non-sealed, bare lands that have unit-soil loss rates that are much higher than comparable rates for undisturbed areas [26,27]. The urban road network is also a major source of sediments in urbanized river basins [28]. The binary approach was also applied to assess the environmental risk of erosion (Table 2).

If a building were affected, the residents might lose their lives, or would be in danger of being trapped in their houses and cut off from public transportation, social infrastructure and health care. In addition, they could suffer damage from extreme floods due to psychic trauma, stress and contaminated drinking water [29]. The indicator of affected population per residential building was quantified by using population-density data at a spatial scale of single houses. The average population per household in the study area was 2.63 [22]. The residential buildings were classified into four types (Table 3) based on surveys. It was assumed that all of the buildings of one type had the same number of floors and households. By assigning a population to the corresponding residential buildings and dividing the value by the area of the first floor, a final map of the population density of residential buildings was produced. By intersecting this population density map with the map of inundation extent, the number of affected people could be estimated. In addition to the affected population, the number of affected social hot spots was used as an indicator to estimate social risk (Table 3). This indicator was also allocated using the binary approach.

### 3.3. Probability Estimation

Dam-failure probability can be characterized by event tree analysis [30], which is mostly based on engineering [31]. Event tree analysis was carried out based on the outcomes of a panel discussion. The panel consisted of four experts from the Dam Safety Management Department (MWR). After the discussion of the experts, an agreement on event trees was achieved. To meet the criteria of a .worst-case scenario. of dam failure, the failure event with the largest probability was considered. This was piping failure at check flood (probabilities are shown in Table 4). In addition to flooding caused by dam failure, a flood event with a probability of 0.02 per year was selected according to the flood protection standards of Chuzhou City. By combining the different flood events, the risk of an indicator follows the equation below [32]:

(1) $$R=\sum_{i=1}^{m}D_{i}*P_{i}$$

where *R* is the flood risk of the indicator; *D_i_* is the damage caused by the *i*th flood event with a probability of *P_i_*; and *m* is the number of flood events (including a piping failure and a 50-year flood) in the case study.

### 3.4. Flood Inundation Modeling

**Figure 4.**
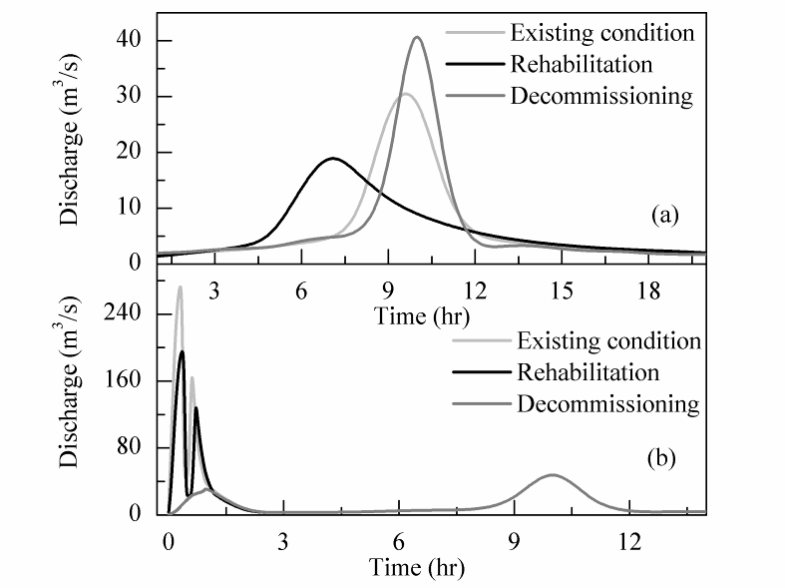
Upstream boundary conditions for inundation modeling of **(a)** 50-year flood events and **(b)** dam failure floods.

To quantify the indicators shown in Table 2, the prime physical parameters of flood inundation (e.g., depth and extent) are needed. The common approach to urban-flood modeling is to employ a 2D approach at high resolution and to calibrate the friction parameters to the observed data [33]. In the present study, the MIKE21 Flow Model [34] was used to model inundation depth. The data that were used to carry out the simulation included the following:

(1) Boundary conditions. The upstream boundary conditions were dam-outflow hydrographs. If the dam is decommissioned, the reservoir will have no flood-control capacity (Table 1); thus, its inflow hydrograph (Figure 4a) was set as an upstream boundary condition for the modeling of a 50-yr flood event.

For the existing condition and the rehabilitated dam scenario, outflow hydrographs were calculated employing the higher-order Runge-Kutta reservoir routing method [35]. The calculated results are shown in Figure 4a. A piping failure of the Heiwa Dam was simulated using the BREACH model [36]. Parameters describing the physical characteristics of the dam were used to obtain an outflow hydrograph of dam failure (Figure 4b). The initial water-surface elevation for a piping breach reflected a normal operating elevation (Table 1); the piping inflow was equal to the 100-year flow rate as the check flood event. The downstream boundary condition was set to a constant discharge of 8 m^3^/s, which is equal to the drainage capacity of a pumping station located at the outlet of watershed (Figure 2).

(2) Terrain data. To replicate the high spatial-height variability of urban areas, topographic input for modeling was considered as two distinct layers, with a mask of building locations and elevations overlaying the ‘bare earth’ terrain [37]. A bare earth DEM was obtained from a 1:10,000 contour map that had a 1-m vertical interval for flat areas and a 2.5-m interval for steeper areas. Building heights were inserted into the bare earth DEM based on their locations, which were obtained from a land use map. Choosing the appropriate model resolution is important for urban flood inundation modeling; the grid resolution was set to 10 m according to the dimensions and separation of the buildings [37].

### 3.5. Decision Rule and Criteria Weighting

A weighted summation (WS) was used to combine the different judgments to reach a final overall ranking of scenarios. WS is the most simple and widely applied MCDA technique [38]. It first transforms all of the indicators into a commensurate scale between 0 and 1 by linear standardization as follows:

(2) $$v_{\text{jk}}=\frac{R_{\text{jk}}-{\text{min}}_{j}(R_{\text{jk}})}{{\text{max}}_{j}(R_{\text{jk}})-{\text{min}}_{j}(R_{\text{jk}})}$$

where *R_jk_* is the risk from the *k*th scenario for the *j*th indicator; *v_jk_* is the standardized value of *R_jk_*; and min*_j_*(*R_jk_*) and max*_j_*(*R_jk_*) are the lowest and highest risk values, respectively, for the *j*th indicator across all of the scenarios. It then sums the indicators multiplied by weights, as follows:

(3) $$R_{k}=\sum_{j=1}^{n}v_{\text{jk}}\times w_{j}$$

(4) $$\sum_{j=1}^{n}w_{j}=1$$

where *R_k_* is the multicriteria flood risk of the *k*th scenario; *w_j_* is the weight of the *j*th indicator; and *n* is the number of criteria.

Structured interviews [39] were carried out among local citizens, water resource managers and dam safety engineers to incorporate the opinions of different stakeholders regarding criteria weights. Weights of key criteria were elicited by a direct rating method [40]. For each indicator, respondents were asked to directly give a rating (out of 100) according to its relative importance in analyzing flood risk [40]. For the *j*th indicator, the weight (*w_j_*) given by an individual was calculated by Equation (5) as follows [40]:

(5) $$w_{j}={\text{IL}}_{j}/\sum_{j=1}^{n}{\text{IL}}_{j}$$

where *IL_j_* is the rating score of the *j*th indicator given by the individual and *n* is the number of indicators. The results of the weight sets are shown in Table 5.

### 3.6. Sensitivity Analyses of Criteria Weights

Indicator weights are often uncertain for two reasons: (1) decision makers may be not aware of their preferences regarding the indicator; and (2) multiple stakeholders have different opinions. Because these weights are usually based on highly subjective judgments, the stability of the priority under varying indicator weights must be tested. Each indicator weight was altered for the increment of value of *a*, as follows:

(6) $$w(c,q)=w_{\text{min}}+a\times q,\;w_{\text{min}}/a\leq q\leq w_{\text{max}}/a$$

where *c* is the main changing indicator under consideration; *w* (*c*, *q*) is its weight at the *q*th change; and *w*_min_ and *w*_max_ are its minimal and maximal weights, respectively. The weights of other indicators were adjusted proportionally to satisfy the additivity constraint in Equation (4), as follows:

(7) $$w(c_{j},q)=[1-w(c,q)]\times w(c_{j},0)/[1-w(c,0)],\;1\leq j\leq n\;\text{and}\;c_{j}\neq c$$

where *w*(*c_j_*, *q*) is the weight of the *j*th indicator; *w*(*c_j_*,0) and *w*(*c*,0) are the mean weights of the *j*th indicator and the main changing indicator under consideration, respectively; and *n* is the number of indicators.

## 4. Results and Discussion

### 4.1. Calibration Results of Flood Inundation Modeling

**Figure 5.**
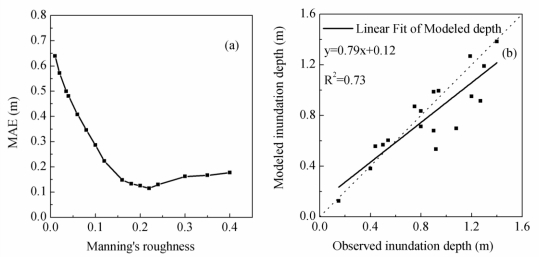
**(a)** MAE of different values of Manning’s roughness from 0.01 to 0.4; **(b)** comparison of the observed and modeled inundation depth using Manning’s roughness of 0.22.

The most detailed data observed in the study were the inundation depth of 18 points (Figure 2) in the flood event that occurred on August 1, 2008. This event, which had an estimated return period of 30–100 years [41], was caused by a rainstorm that generated up to 429 mm of rain in approximately 24 hours [42] and led to the inundation of various localities with water depths of more than 1.5 m, causing extensive property damage [41]. To calibrate the roughness coefficient of these observed data, 16 simulations were conducted with the Manning’s n values varying from 0.01 to 0.4 [33,37,43]. The response surface (Figure 5a) suggests an improvement in model performance at high values of Manning’s n, but values larger than 0.22 decreased model performance. Consequently, a uniform Manning’s n of 0.22 was used.

In an urban area, a prevailing approach for predicting flood inundation is dual-drainage modeling, which combines a 2D overland-flow routing model, a 1D channel-flow model and a sewer system model [44,45]. For the case study of the Heiwa Dam, a simplification to 2D overland flow is reasonable for several reasons. Firstly, the small natural channels have been encroached upon and filled during the progress of urbanization and thus have lost their ability to drain floodwater. Secondly, the discharge capacity of the sewer system has a maximum value of 5 m^3^/s, which is approximately 10% of the peak discharge in a 50-year flood under existing conditions (Figure 4a); therefore, the influence of the sewer system was ignored in this study. The comparison of modeled and observed inundation depths showed that *R*^2^ was 0.73 (Figure 5b), which indicated that the performance of the model was acceptable.

### 4.2. Flood Risk under Existing Conditions

**Figure 6.**
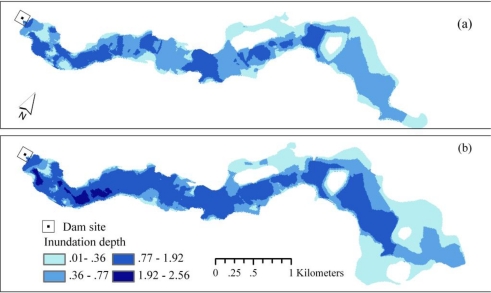
Modeled maps of inundation depths in **(a)** 50-year floods and **(b)** dam-failure floods under existing conditions.

In the case study, the elevations of the first floors of buildings were generally the same as those of the surrounding territory, and there were no protective measures; thus, it was reasonable to assume that the damaging water depth inside the buildings was the same as the water depth across the terrain [14]. The spatial patterns of damaging water depth during 50-year and dam-failure floods under existing conditions are shown in Figures 6a and b. These figures reveal that the flood zone of the dam-failure event was approximately 3.3 km^2^, which was approximately 37.5% larger than that of a 50-year flood event. The enlargement area of the flood was primarily located in a rural area to the east of the Jinghu Railway (Figure 2). The urban areas located downstream of the dam site and west of the Jinghu Railway had a slight enlargement in the extent of flooding but experienced a significant increase in the inundation depth compared with the 50-year event (Figures 6a and b). The most seriously inundated areas were those near the dam site (including the Shanshui Residential Community and the western part of the Chuzhou High-tech Zone along Fengle Road; Figures 2, 6a and b) where the water depth was deeper than 0.77 m during a 50-year flood event and reached 2.56 m if the dam failed. These areas are densely populated or have a high concentration of businesses and are thus vulnerable to flooding. These results reflect the fact that the flood inundation risk for the urban areas downstream of the dam site is serious under existing conditions.

**Figure 7.**
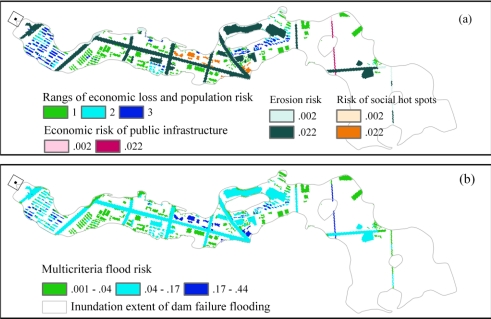
**(a)** Flood risk of each indicator and **(b)** multicriteria flood risk under existing conditions. Economic-loss risk (yuan/year/cell) and population at risk (person/year/cell) were classified into the three following ranges: 1 (2 < economic loss risk < 100 or population at risk < 0.002); 2 (100 ≤ economic loss risk < 228 or 0.002 ≤ population at risk < 0.007); and 3 (228 ≤ economic loss risk ≤ 490 or 0.007 ≤ population at risk < 0.02).

For each of the five indicators, a separate risk map of the existing condition was computed based on damaging water depths. The spatial patterns of economic-loss risks and populations at risk in residential buildings overlapped, so the risks of the two indicators were combined and classified into three ranges (Figure 7a). The highest risk (economic loss risk > 228 yuan/year/cell; population at risk > 0.007 person/year/cell) was found in the residential areas (Figure 7a) including Shanshui and the Bali Residential Community (Figure 2). Low economic-loss risk (values from 2 to 100 yuan/year/cell) was distributed in the western and northern parts of the Chuzhou High-tech Zone along Fengle Road (Figures 2 and 7a). The social hot spots, including the municipal administration center and the blood bank (Figure 2), were affected in two flood events (Figures 6a and b); hence, the inundated probability per year was 0.022 (Figure 7a). Most of the inundated areas with erosion potential were also affected in both of the flood events and thus had erosion risks of 0.022. A section of the Jinghu Railway (Figure 2) of approximately 600 m might also be inundated (with a probability of 0.022 per year). The areas without flood risk (e.g. that are not vulnerable to flooding) include impervious areas (except roads) and submergence-tolerant grassland.

When the flood risks of the five indicators were summed using the mean weight sets (Table 5), the multicriteria flood risk was calculated (shown in Figure 7b). High multicriteria flood risks (values from 0.17 to 0.44) were found in portions of the Shanshui Residential Community, in social hot spots and along a section of the Jinghu Railway.

The elements of medium-level risk (values from 0.04 to 0.17) were distributed over the residential buildings, main roads and bare lands in the inundated areas. The commercial buildings had the lowest multicriteria risk (<0.04).

### 4.3. Changes in Multicriteria Flood Risks of Two Scenarios

**Figure 8.**
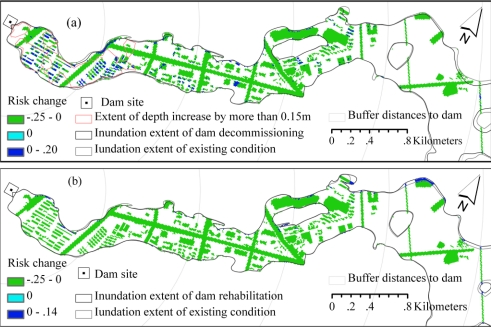
Changes of multicriteria flood risk from risk under existing conditions in two scenarios: **(a)** dam decommissioning scenario and **(b)** dam rehabilitation scenario.

The multicriteria flood risks of the two scenarios were also computed using the mean weight sets shown in Table 5. The spatial patterns of the changes in multicriteria risk from risk under existing conditions are shown in Figure 8. In the dam decommissioning scenario, areas with increased risk (<0.20) were found in some of the residential and commercial buildings (e.g., those in which the inundation depth was increased by more than 0.15 m during a 50-year flood event; Figure 8a). Other elements in the flood zone had risks that were decreased by up to 0.25 (Figure 8a) because the dam-failure probability was significantly reduced when the dam was decommissioned (Table 4). The dam rehabilitation scenario reduced the risk by up to 0.25 in the most affected areas as a combined result of the lower water depth during a 50-year flood and the smaller probability of dam failure (Table 4). However, areas at the fringes of the inundation zone were found to have increased risk because the dam rehabilitation alternative slightly enlarged the extent of inundation (Figure 8b).

**Figure 9.**
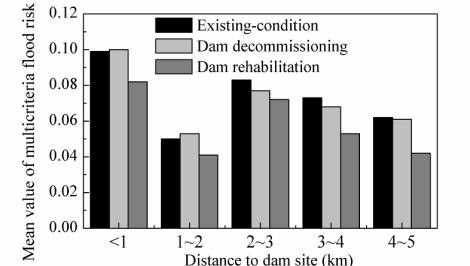
Comparison of multicriteria flood-risk averaged over different distances to the dam site among three scenarios.

In the dam decommissioning scenario, the mean risk near the dam site (e.g., at a distance of less than 2 km; Figure 9) was larger than the risk under existing conditions as a result of the increased risk to residential and commercial buildings (Figure 8a). As the distance to the dam site increased, the mean flood risk decreased (as compared with the existing conditions). The dam rehabilitation scenario always had a smaller mean risk, even in areas less than 1 km downstream of the dam site (Figure 9).

### 4.4. Weight Sensitivity

**Figure 10.**
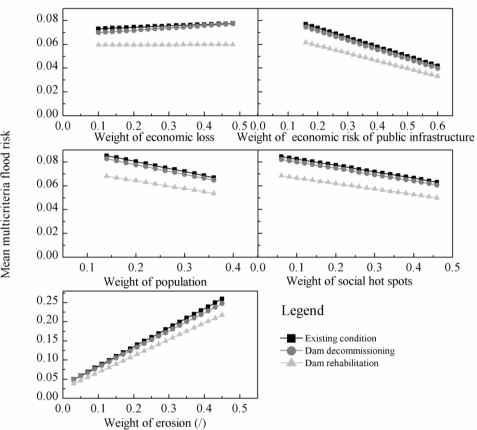
Sensitivity analyses results: the changes of mean multicriteria flood risk for each alternative with an increment (increasing or decreasing by 0.02) of the weight of each individual indicator.

The sensitivity analyses were carried out by using an incremental change (0.02) in the weight of each individual indicator. The multicriteria map was translated into a non-spatial multicriteria analysis by spatial aggregation. The most commonly used method is to take the average of the values in the map [18]. After spatial aggregation, the mean risk values of the cells are used to rank the ability of the scenarios to reduce the multicriteria flood risk (Figure 10). The most sensitive relative indicators were *erosion* and *economic loss*, which resulted in the ranking of the dam-decommissioning scenario at a level very close to the ranking of the existing conditions. The perturbation of the weights in other indicators (particularly *social hot spots*) had a slight impact on the rankings. This revealed that the rankings were almost independent of changes in the weights associated with this indicator; thus the ranking was robust [15]. Although the results obtained from the sets of weights displayed some differences, the general structure of the final rankings was similar: the rehabilitation scenario placed first, the decommissioning scenario was second, and the existing conditions tended to be in the last position (Figure 10).

### 4.5. Scenario Appraisal and Recommendations

Despite variation in the indicators weights, the dam rehabilitation scenario maintained the highest ranking. This scenario decreased the mean risk by no more than 23% over the risk under existing conditions (Figure 10), yet this decrease was small when compared with its resultant increase (45%) in the reservoir flood-control storage capacity (Table 1). These results indicate that the aging-dam management scenario alone (which increases the flood-control capacity of the dam) can be of little help in the flood-risk abatement effort examined in this case study. Therefore, a comprehensive plan addressing flood-risk management is necessary. Dewan *et al.* [46] suggested that this plan should combine land-use strategies with the careful consideration of certain structural controls. They emphasized the preservation of natural channels and infiltration processes during urban planning. In the case of Chuzhou city, however, small channels were often encroached upon during the urbanization progress and thus lost their ability to drain floodwater. It is crucial to improve the drainage systems of the city and to prevent the further filling in of channels to dramatically decrease the flood risk.

The ranking of dam decommissioning was generally in the middle, although some exceptions remained for the indicators of *economic loss* and *erosion*. If maximal and minimal weight values, respectively, were assigned to the two indicators, the mean risk of the dam-decommissioning scenario ranked very close to the risk under existing conditions. When the weight of *economic loss* reached 0.48, the mean risk of the dam-decommissioning scenario increased almost to that of existing conditions (Figure 10). The dam-decommissioning scenario increased the economic-loss risk for some of the residential and commercial buildings (Figure 8a); thus, the larger weight assigned to *economic loss* enlarged its disadvantage. When a minimal weight was given to *erosion*, the mean risk of the decommissioning scenario was as large as the risk under existing conditions. In general, the mean risk of all of the cells is not greater than the risk under existing conditions if the dam is decommissioned (although areas with increased risk were found in some residential and commercial buildings). It seems that the flood-control capacity of the Heiwa Dam is limited, and decommissioning it will not significantly increase the flood risk. The priority of the dam-decommissioning scenario could be increased if a comprehensive plan addressing flood-risk management (i.e., improving the drainage systems of the city) were to be implemented. The priority of the dam decommissioning scenario may be further increased considering its beneficial effects on ecosystem restoration [47].

### 4.6. Potentials and Limitations of the Framework Adaptation

The framework presented here shows the multidisciplinary characteristics of analyzing multicriteria flood risks in aging-dam management. It involved the research regarding dam-safety engineering, hydraulic, hydrological, environmental, economic and social issues. Furthermore, the participants included relevant stakeholders, experts, and decision makers. The multidisciplinary and multiple-participant characteristics of the study made it possible to incorporate dam-safety management with flood-risk management.

It should be noted that this framework is a conceptual one. It can be applied to other locations, but its specific methods should be adjusted or improved according to the circumstances of each case and the availability of data. In particular, the criteria should be augmented when more detailed data are available. The binary approach, which only considers the influence of the flood inundation extent on risk, should be enhanced when more spatially explicit quantitative data are available (along with the development of urban statistics and census works). The methods to predict the probability of dam failure, the flood-inundation model, and the MCDA rules should also be adjusted accordingly.

## 5. Conclusions

Developing a framework for the analysis of spatial multicriteria flood risk that incorporates economic, social and environmental dimensions is crucial for decision making for dam management, especially in urban areas. Based on spatial multicriteria decision analysis theory, a framework was developed to analyze the changes in the multicriteria flood risk of two management scenarios with regard to risk under existing conditions. This framework included scenario definition, problem structuring, criteria construction, spatial quantification of criteria, criteria weighting, decision rules, sensitivity analyses, and scenario appraisal. It is a conceptual framework that can be applied to other places, and its specific methods and criteria should be adjusted or improved according to the circumstances of each case and the availability of data.

In the case study of the Heiwa dam, the existing-condition analyses following this framework indicated that there would be serious flood inundation even in a 50-year flood event. A high flood risk was found for some of the residential buildings, social hot spots and the Jinghu Railway. The dam-rehabilitation simulation indicated that the multicriteria flood risk was reduced by 0.25 in most of the affected areas and resulted in a reduction of the mean risk. However, this reduction was no more than 23% based on the sensitivity analysis. If the dam were to be decommissioned, an increase in the inundation depth of more than 0.15 m would be found near the dam site where residential and commercial buildings are concentrated. This would cause the multicriteria flood risk to be increased by 0.20 in that location. However, the less intense and less frequent dam-failure flooding that follows dam decommission did not make the mean risk greater than the risk under existing conditions.

These results indicated that in the case study (and under the current flood management system) the dam rehabilitation scenario had a higher rank for decreasing the flood risk than did the decommissioning scenario. However, the dam rehabilitation scenario might be of little assistance to efforts of flood-risk abatement. A comprehensive plan addressing flood-risk management is necessary. Such a plan could efficiently diminish the flood risk for urban areas and improve the priority of the dam-decommissioning scenario. The ranking of the dam-rehabilitation and decommissioning scenarios with the implementation of such a plan should be further analyzed.

## Figures and Tables

**Table 1.** Summary of the key physical characteristics of the management scenarios.

**Scenario**	**Max. dam height/m**	**Normal operating depth/m**	**Storage capacity/10^4^ m^3^**		
			**Normal depth**	**Flood control**	**Total**
Existing-condition	12.2	10	44.5	11.5	56
Decommissioning	4	2	9	0	9
Rehabilitation	12.2	8.8	35.3	16.7	52

**Table 2.** Indictor sets for multicriteria flood-risk analyses in the case study.

**Criteria**	**Indicator**	**Indicator description**	**Damage unit(…/cell)**
Economic	Economic loss	Loss to assets and inventories of residential, industrial, and commercial buildings	Yuan
	Economic risk of infrastructure	Affected public infrastructure such as railways, telephones and electricity	Binary:
Environmental Social	Erosion	Affected non-sealed areas and major urban roads	inundated = 1;
	Social hot spots	Affected social infrastructure such as schools, kindergartens, retirement homes, and hospitals	un-inundated = 0
	Population	Affected population per residential building	Number of people

**Table 3.** Classification of building heights and households.

**Residential buildings**	**Number of floors**	**Number of households**
Multi-story buildings	4–7	16–18
High-rise buildings equipped with elevators	8–13	48–78
Low-rise buildings	1–3	1–6
Rural buildings	1–3	1

**Table 4.** Results of event-tree analysis of piping failure.

**Scenario**	**Probability of events**					
	**Flood**	**Infiltration of downstream slope**	**Concentrated seepage**	**Piping**	**Intervention failure**	**Dam failure**
Existing-condition	0.01	0.9	0.5	0.9	0.5	2.03E-03
Decommissioning	0.01	0.1	0.1	0.1	0.1	1.00E-06
Rehabilitation	0.01	0.1	0.1	0.1	0.5	5.00E-06

**Table 5.** Weight sets of indicators.

**Criteria**	**Indicator**	**min**	**mean**	**max**
Economic	Economic loss	0.1	0.21	0.48
	Economic risk of public infrastructure	0.16	0.19	0.6
Social	Population	0.14	0.27	0.36
	Social hot spots	0.06	0.25	0.45
Environmental	Erosion	0.03	0.08	0.44
